# Capecitabine-Induced Ileitis during Neoadjuvant Pelvic Radio-Chemotherapy for Locally Advanced Rectal Cancer: A Case Report with Literature Review

**DOI:** 10.3390/curroncol30100655

**Published:** 2023-10-10

**Authors:** Andrea Brignoli, Eleonora Ferrara, Micol Zannetti, Gianfranco Loi, Laura Forti, Carlo Socci, Alessandro Carriero, Alessandra Gennari, Marco Krengli, Pierfrancesco Franco

**Affiliations:** 1 Division of Radiation Oncology, University Hospital “Maggiore della Carità”, 28100 Novara, Italy; andrea.brignoli@maggioreosp.novara.it (A.B.); eleonora.ferrara@maggioreosp.novara.it (E.F.); micol.zannetti@maggioreosp.novara.it (M.Z.); 2 Department of Translational Medicine, University of the Eastern Piedmont, 28100 Novara, Italy; alessandro.carriero@med.uniupo.it (A.C.); alessandra.gennari@med.uniupo.it (A.G.); 3 Department of Medical Physics, University Hospital “Maggiore della Carità”, 28100 Novara, Italy; gianfranco.loi@maggioreosp.novara.it; 4 Division of Medical Oncology, University Hospital “Maggiore della Carità”, 28100 Novara, Italy; laura.forti@maggioreosp.novara.it; 5 Division of Surgery, Hospital ‘S.S. Trinita’, 28021 Borgomanero, Italy; carlo.socci@asl.novara.it; 6 Division of Radiology, University Hospital “Maggiore della Carità”, 28100 Novara, Italy; 7 Radiation Oncology, Veneto Institute of Oncology IOV-IRCCS, 35100 Padua, Italy; marco.krengli@unipd.it; 8 Department of Surgery, Oncology and Gastroenterology, University of Padova, 35100 Padua, Italy

**Keywords:** rectal cancer, radiation therapy, ileitis, capecitabine, concurrent radiochemotherapy

## Abstract

We report on a clinical case of capecitabine-induced acute ileitis in a patient treated with pre-operative concurrent chemoradiation with capecitabine for locally advanced rectal cancer and provide a comprehensive literature review. This a rare, but life-threatening, clinical situation, that clinicians should be aware of. Severe persistent diarrhea is the most frequent clinical feature and computed tomography is a valid tool for diagnosis. Conservative management includes capecitabine withdrawal, antidiarrheal therapy and endovenous hydration, together with dietary modifications and broad-spectrum antibiotics. Pelvic irradiation represents an adjunctive risk factor, which may increase the likelihood of occurrence of terminal ileitis. Early recognition and prompt intervention are crucial for successful clinical management.

## 1. Introduction

Colorectal carcinoma ranks third in terms of prevalence in the Western world, and the rectal subsite alone contributes to over 700,000 new cancer diagnoses each year, for a relative percentage of around 30% [1]. The aim of preoperative treatments for locally advanced rectal cancer is to optimize local control, achieve tumor downsizing and downstaging and possibly pathological complete response, increase the rate of circumferential margin-free surgical resections, facilitate the feasibility of sphincter-preserving surgery and improve disease-free survival; it may also lead to clinical complete remission, allowing organ preservation [2,3,4,5]. Concurrent treatments often involve the combination of radiation therapy (RT) and capecitabine, an oral precursor of 5-fluorouracil [2,6]. Radiation enteritis could be a complication of RT for pelvic tumors (i.e., rectum, prostate, cervix, endometrium) and can affect both large and small bowel [7]. Common clinical symptoms are diarrhea, abdominal pain and nausea. Capecitabine is associated with several side effects, including mucositis, hand-foot syndrome, nausea, vomiting, and general malaise. In addition, more serious side effects, such as bowel obstruction and bowel perforation have been reported [8]. Hereby, we present a case of acute ileitis in a patient with locally advanced rectal cancer who underwent preoperative concurrent radiochemotherapy (RT-CHT) with capecitabine. We also provide a comprehensive review of the relevant literature.

## 2. Detailed Case Description

### 2.1. Clinical Case Presentation

A 71-year-old woman, with a clinical history of hypertension and hysteroannessiectomy for uterine fibromatosis, presented with symptoms of rectal bleeding, increased bowel frequency, and weight loss. She underwent a full colonoscopy which revealed an ulcerated and vegetating lesion in the mid-lower rectum (5 cm from the anal verge). Biopsies confirmed the presence of adenocarcinoma. The patient completed her oncological staging with computed tomography of the chest and abdomen, which excluded the presence of metastasis, and a pelvic magnetic resonance (MR) scan. The MR scan confirmed the presence of a lesion in the mid-lower rectum with a cranio-caudal extension of 6 cm, infiltrating the full thickness of the rectal wall and perivisceral fat by less than 5 mm. Multiple lymph nodes with pathological features were observed in the mesorectal fat; the mesorectal fascia and extramural vessels were found to be uninvolved (stage IIIB; cT3b cN2 MRF negative; extramural vascular invasion negative, according to AJCC/UICC TNM staging system, 8th edition) [9]. Based on the baseline clinical stage, the initial treatment approach offered to the patient was ‘total neoadjuvant therapy’, with preoperative RT treatment combined with concurrent capecitabine and subsequent consolidation chemotherapy. For concurrent RT-CHT, the prescribed RT dose to the elective pelvic nodal groins (bilateral internal iliac, presacral and obturator nodes) was 45 Gy in 25 fractions (1.8 Gy daily), with a simultaneous integrated boost up to 50 Gy (2 Gy daily) to the macroscopic disease and corresponding mesorectum, identified as the high dose Clinical Target Volume (CTV) [10,11]. An 8 mm isotropic margin was added to the CTV to create the corresponding Planning Target Volume (PTV). The treatment was delivered using a volumetric intensity modulated technique (VMAT) with 6 MV-X rays generated by a Varian Trilogy Tx linear accelerator.

**Figure 1:**
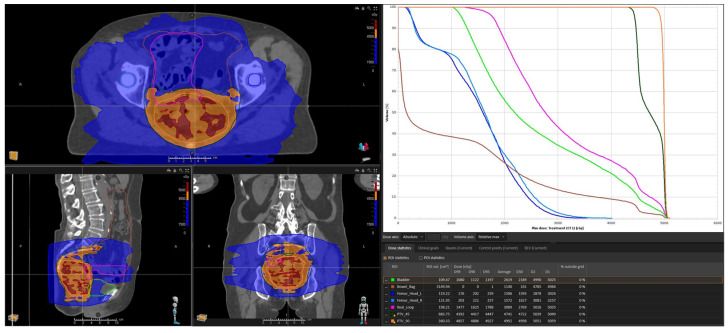
Dose distribution and dose-volume histogram of the case reported. On the bowel loops that resulted in being fixed (highlighted in pink), the V_15_ (<120 cc) and V_45_ (<15%) constraints could not be satisfied, due to the risk of underdosage on the high dose PTV. The 15 Gy, 45 Gy and 50 Gy isodose curves are highlighted in blue, orange and red, respectively.

The patient underwent treatment simulation in the supine position, and the computed tomography scan revealed a few bowel loops located in close proximity to the high-dose CTV. A VMAT plan was optimized reducing the dose to these loops as best achievable, with the objective of full CTV coverage at the prescribed dose (Figure 1). The maximum dose objectives of V_15_ (<120 cc) and V_45_ (<15%) on a single loop were not satisfied, while those for V_50_ on a single loop (<10 cc) and V_45_ on the entire bowel bag (<195 cc) were met (as reported in Table 1) [12]. Treatment delivery was monitored by image guidance with Cone Beam CT (CBCT). Concurrent CHT with capecitabine at an 825 mg/m^2^ twice-a-day dose (total daily dose of 2500 mg) was started on day 1; a dihydropyrimidine dehydrogenase (DPD) test was performed prior to initiation of treatment, which revealed a wild-type genotype. During the first 15 fractions, the patient showed no signs of gastrointestinal toxicity, and weekly blood tests (including blood count, and renal and liver function) were within normal range.

**Figure 2:**
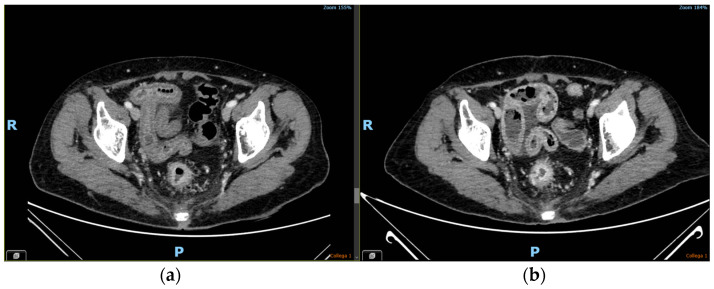
Abdominal computed tomography scans performed at the onset of the symptoms (**a**) and 12 days later (**b**); the former shows a massive ileal wall thickening with hyperemia of the mucosa and perivisceral vascular enhancement, findings that can still be observed (although in reduction) in the latter.

At fraction 17 (total radiation dose: 34 Gy to the macroscopic disease and 30.6 Gy to the elective volumes; total capecitabine dose: 42, 500 mg), the patient reported multiple episodes of watery diarrhea, which did not respond to loperamide, combined with nausea (no vomiting), and diffuse abdominal pain (grade 3 acute gastrointestinal toxicity according to Common Terminology Criteria for Adverse Events, version 5.0) [13]. Upon physical examination, the patient complained of severe abdominal pain that was exacerbated by palpation but did not display any sign of peritonism. Hematological analyses were performed, which unveiled the presence of G1 leukopenia (WBC: 3120/μL), a slightly elevated level of C-reactive protein (1.4 mg/dL, with a normal threshold of <1 mg/dL), and mild hypokalaemia (potassium: 3.4 mEq/L, with a reference range of 3.5–5 mEq/L). A diagnostic computed tomography scan of the abdomen showed thickened ileal walls, with mucosal hyperemia and vascular enhancement, as illustrated in Figure 2a. All these findings supported the diagnosis of an inflammatory clinical condition.

Subsequently, the patient was admitted to the radiation oncology inpatient ward, where a stool analysis was performed which turned negative. A prophylactic broad-spectrum antibiotic regimen, consisting of ciprofloxacin 400 mg b.i.d. and metronidazole 500 mg t.i.d., was administrated. The patient received supportive therapy which involved bowel rest, caloric intake through parenteral nutrition, intravenous rehydration, and anti-inflammatory treatment with oral budesonide. The concurrent RT-CHT treatment was halted. Following a period of six days of bowel rest and considering the patient’s positive clinical progress, oral food intake was gradually resumed, with good tolerance. On the twelfth day following admission, an abdominal computed tomography scan was repeated, revealing a minor reduction in bowel wall edema as depicted in Figure 2b. Following a period of 20 days, the patient had a complete resolution of diarrhea and abdominal pain, which enabled her to intake a low-residue diet without the requirement of parenteral supplementation. Laboratory findings went back to normal values (WBC: 6860/μL, potassium: 3.9 mEq/L, C-reactive protein < 1 mg/dL). Consequently, she was discharged from the medical facility. However, due to the severe toxicity and prolonged treatment suspension, the concurrent RT-CHT was not resumed and the initial program of ‘total neoadjuvant therapy’ was dropped.

**Figure 3:**
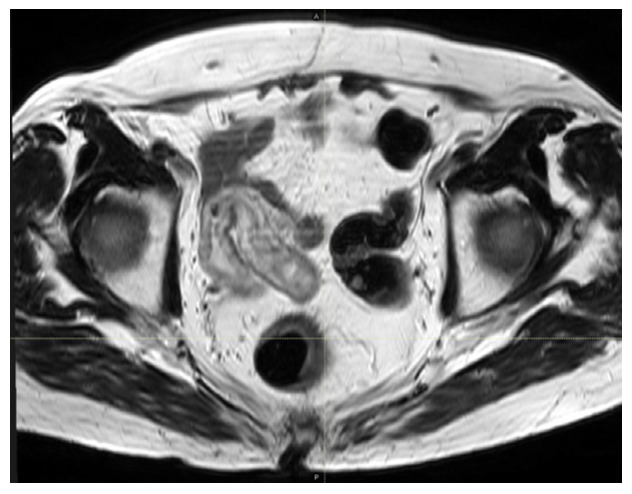
Pelvic MRI scan performed 6 weeks after the interruption of the radiochemotherapy. Compatibly with the different exam, a moderate thickening of the ileal walls can still be noticed.

Six weeks after the interruption of concurrent RT-CHT, a pelvic MRI was performed to restage the disease prior to surgery which, compatibly with the different diagnostic methods, collaterally showed a persistence, although reduced in severity, of the ileal walls edema (as shown in Figure 3). Thereafter, the patient underwent an anterior rectal resection with total mesorectal excision, which, according to the pathological report, showed a partial response to the preoperative treatment (ypT2 ypN0, with negative surgical margins). After multidisciplinary discussion, adjuvant capecitabin was initiated at lower doses, with close monitoring of the potential gastrointestinal toxicity. The patient completed six cycles of adjuvant capecitabine at a total daily dose of 3000 mg (reduced to 80% accounting for the previous severe toxicity), with no major gastrointestinal and hematological events. Six months after completion of adjuvant chemotherapy, she is alive and free from disease.

### 2.2. Search Strategy and Data Extraction

The literature search strategy addressed colorectal and rectal cancer patients (population) undergoing treatment with capecitabine (intervention), who experienced ileitis (outcome). The search was carried out and finally updated on 31 March 2023. All studies published between 1 January 2000 and 31 December 2022, reporting on the outcomes of interest in the selected population were identified. The search terms ‘colon cancer’, ‘rectal cancer’, ‘capecitabine’, ‘radiotherapy’ and corresponding synonyms were employed to search PubMed/MEDLINE, Embase and Cochrane Library databases. All articles were then combined into a single list, and duplicates were excluded. Studies were included in the present review if they reported on the outcome of interest (ileitis) in colorectal cancer patients undergoing capecitabine-based treatments. Publications written in languages other than English were excluded. Finally, reference lists of included articles were screened for other potentially relevant articles. One co-author (AB) extracted the data regarding the clinical case, patient characteristics, type of treatments, clinical data, and outcomes from each publication. The selection of studies is reported in Supplementary Material Figure S1.

## 3. Discussion

The complications associated with pelvic RT are influenced by several factors, including the extension of the treatment volume, the cumulative dose and fractionation schedule, the intensity of the radiation, and the specific treatment approach employed. Bowel toxicity is directly related to the volume of small intestine irradiated [14]. More conformal techniques, such as Intensity Modulated Radiation Therapy (IMRT) and VMAT, can effectively reduce the volume of the small bowel receiving medium to high doses. The loss of intestinal crypt epithelial cells is responsible for the direct radiation damage to the small bowel mucosa. The depletion of these proliferating cells leads to epithelial atrophy, which in turn results in the malabsorption of different substances (fatty acids, carbohydrates, and proteins). This also allows luminal microbes and their byproducts to reach innate immune cells in the lamina propria, triggering an immune response. Additionally, damage to endothelial cells due to radiation can also significantly contribute to the development of radiation-induced intestinal damage [15]. Combined modality approaches are associated with an increased risk of bowel toxicity. The addition of 5-fluorouracil to pre-operative RT acts as a radiosensitizer, resulting in a notable improvement in local control [16]. Nevertheless, this combination increases the incidence of acute intestinal toxicity, characterized by symptoms such as nausea, diarrhea, and abdominal pain [17,18]. The study conducted by the European Organization for Research and Treatment of Cancer in 2006 revealed that the addition of CHT to RT in the pre-operative treatment of rectal cancer resulted in an increased incidence (17% vs. 38%) and severity of diarrhea [16]. The increased susceptibility to toxicity may be attributed to the modifications in the cell cycle kinetics and the synchronization of replicating cell populations. Halopyrimidines, such as fluorouracil, act by inhibiting effective DNA repair and increasing the amount of radiation-induced DNA damage [15]. The conversion of capecitabine through gastrointestinal absorption and enzymatic metabolism results in the generation of 5-fluorouracil, which is the pharmacologically active constituent of the medication [6]. The main advantages of capecitabine are its oral administration and a favorable tolerability profile. The manifestation of adverse events during capecitabine therapy depends on the regimen and dosage of the medication. Most of these adverse effects can be efficiently managed through dose reduction or discontinuation of treatment. Nevertheless, specific adverse effects may present a substantial hazard to a patient’s well-being and necessitate hospitalization [7]. The etiology of capecitabine-induced ileitis remains unclear; however, literature evidence suggests that two primary mechanisms may co-operate to its onset: (a) the ileal mucosal epithelial cells exhibit a cytotoxic response; (b) fluoropyrimidines are known to cause vasoconstriction, leading to a decrease in mucosal blood flow [19,20]. We reported a case of acute ileitis observed in a patient who received a diagnosis of locally advanced rectal cancer and subsequently underwent concomitant RT-CHT with capecitabine. The literature reports a total of eighteen other cases of ileitis induced by capecitabine, which are fully reported in Table 2 [21,22,23,24,25,26,27,28,29,30,31,32,33,34]. No clearly identifiable risk factors have been established. No case of fatal outcome or permanent bowel damage has been reported. However, in most cases, the occurrence of ileitis hampered the overall treatment intensity with potential repercussions on the oncological outcomes. Notably, only two of these cases were observed during concurrent RT and systemic treatment [27]. In another case, a patient received pelvic RT with palliative intent and experienced toxicity several weeks after the completion of RT, during capecitabine treatment. [24]. Common symptoms of ileitis include severe diarrhea that is unresponsive to loperamide, abdominal pain, and infrequently, fever. Severe bleeding resulting from ileitis has been documented in two cases [29,31]. The use of abdominal computed tomography as a diagnostic modality is valuable in identifying the characteristic edema and distension of the small bowel associated with this specific condition. In case of uncertainty, an endoscopic examination can serve as a helpful diagnostic tool, allowing for biopsies to be taken, to support the diagnosis.

The present case is the third patient with acute ileitis observed during preoperative RT-CHT for locally advanced rectal cancer. The patient had some bowel loops fixed within the pelvic cavity; this is probably due to the previous hysteroannessiectomy, which could have led to some bowel adhesions. Small bowel prolapse into the pelvic cavity or surgical adhesions that fix intestinal segments can predispose part of the intestine to receive higher doses of radiation [35]. Also, bowel motion, which typically occurs during pelvic and abdominal RT on an inter- and intra-fraction basis, can also cause unintended irradiation of sensitive organs at risk such as the small intestine.

As explained above, during treatment planning optimization, not all planning objectives could be met, due to this specific anatomical alteration. Nevertheless, the dose constraint which was significantly overpassed (V15 ≤ 120 cc; in our case 195.3 cc) would have not been met even on an RT delivery platform that could account for motion with online adaptive strategies.

Nevertheless, the patient did not receive the total dose planned and experienced severe acute toxicity after 17 of the 25 planned fractions (at the total dose of 30.6 Gy to the elective nodal stations and 34 Gy to the macroscopic disease and mesorectum, with the ileal loops receiving a mean dose of 21.2 Gy). Furthermore, the diagnostic CT performed at the onset of symptoms revealed modifications in the mucosa of the small intestine that were present in bowel loops situated outside the pelvic region and the radiation field. Considering all these factors, it is more plausible that RT served as a trigger for symptoms onset in a complex mechanism of co-factors in which CHT probably played the main role since enteritis is a listed adverse event in the capecitabine product information document [36].

However, the combined effect of radiation and capecitabine in the occurrence of ileitis should be carefully considered. It is meaningful that, in our case, ileitis was observed at a capecitabine dose of 825 mg/m^2^ twice a day, significantly lower than the dose used during adjuvant chemotherapy for colon cancer (1000–2500 mg/m^2^ twice a day). In this sense radiation may have had a synergistic effect, lowering the threshold of occurrence for ileitis with exclusive capecitabine. This possibility should be carefully accounted for by clinicians during the clinical management of these complex cases.

The optimal treatment strategy for capecitabine-induced ileitis involves prompt discontinuation of capecitabine administration and implementation of conservative interventions aimed at mitigating the related symptoms. Those interventions encompass hydration, bowel rest via total parenteral nutrition, and pain control. Prophylactic administration of a broad-spectrum antibiotic is advised, while the use of corticosteroids, such as budesonide, may be beneficial in managing the inflammatory process. It is imperative for clinicians to be aware of this medical condition, and act promptly with a tailored approach.

## 4. Conclusions

Acute ileitis is a rare (although probably underreported), but potentially dangerous complication of chemotherapy with capecitabine. Clinicians should be aware of that and suspect its occurrence in case of persistent and severe diarrhea. This condition primarily manifests in patients undergoing capecitabine treatment for colon cancer. In patients with rectal cancer treated with preoperative RT-CHT, ileitis may occur at lower doses of capecitabine due to the possible synergy with RT in the onset of this adverse effect; therefore, special care must be taken when planning and optimizing treatment delivery, using modern RT techniques and approaches [37,38]. Clinicians should be mindful of the potential complication of ileitis when establishing the differential diagnosis in patients who are undergoing neoadjuvant RT-CHT with capecitabine. The diagnosis of ileitis is predominantly established through clinical features and the exclusion of alternative causes, and the recommended treatment approach involves discontinuation of capecitabine, along with supportive measures and alleviation of symptoms.

## Figures and Tables

**Table 1:** Dose constraints, as reported by Bisello et al. [12], compared to those observed in our case.

	Constraints	Our Case
Bowel space	V45 ≤ 195 cc	141.7 cc
Small bowel	-Dmax ≤ 55 Gy -V15 ≤ 120 cc (optimal) -V45 ≤ 15% -V50 ≤ 10 cc (optimal) or ≤ 10% (mandatory)	50.98 Gy 195.93 cc 16% 5.9 cc

**Table 2:** Cases of capecitabine-related ileitis reported in literature.

Reference	Patient	Treatment	Capecitabine Dose	RT Doses/Volumes	Clinical Features	Symptoms Onset	Diagnostic Procedures	DPD Testing	Management	Outcome
Barton, 2006 [21]	54 y.o. man with locally advanced colon cancer	Adjuvant capecitabine	N.R.	/	Diarrhea, abdominal pain	After 3rd cycle	Colonoscopy w/biopsy: ulcerative ileitis with eosinophilic infiltrate	N.R.	Bowel rest with IV nutrition and broad-spectrum antibiotics	N.R.
Bouma, 2011 [22]	73 y.o. man with stage IV colon cancer	Palliative capecitabine/oxaliplatin/bevacizumab	N.R.	/	Abdominal pain, diarrhea, nausea	After 3rd cycle	CT: ileal walls thickening	Not performed	Hydration and capecitabine interruption (restarted at reduced dose after 4 weeks)	Clinical recovery after supportive therapy (duration of treatment not reported)
Radwan, 2012 [23]	67 y.o. man with pT4N0 transverse colon cancer	Adjuvant capecitabine	1000 mg/m^2^ twice daily for the first cycle (increased to 1250 mg/m^2^ from the second cycle)	/	Abdominal pain, reduced appetite, diarrhea and giddiness	After 2nd cycle	Abdominal X-ray: small bowel distension. CT: fluid distended loops and distal ileum walls thickening	N.R.	Broad-spectrum antibiotics, symptomatic therapy and permanent capecitabine discontinuation	Clinical recovery after 2 weeks of supportive therapy
Al-Gahmi, 2012 [24]	65 y.o. man with stage IV rectal carcinoma	Palliative pelvic radiotherapy + sequential capecitabine/oxaliplatin	1500 mg/m^2^ twice daily d1-14q21	30 Gy/10 fx on gross rectal disease	Abdominal pain, diarrhea, vomit, fever	12 days after CHT start	Colonoscopy w/biopsy: terminal ileum ulceration with eosinophilic infiltrate	Negative (tested afterwards)	Hydration, broad-spectrum antibiotics and capecitabine interruption (restarted at reduced dose after 5 weeks)	Clinical recovery after CHT discontinuation and supportive therapy. (duration of treatment not reported)
Mokrim, 2014 (case 1) [25]	66 y.o. woman with stage IV breast cancer	Palliative capecitabine	1250 mg/m^2^ twice daily	/	Diarrhea, fever, vomit, fatigue	During 2nd cycle	CT: ileal walls thickening. Colonoscopy w/biopsy: eosinophilic infiltrates	Positive for (DPYD *5,6) mutation (tested afterwards)	Broad-spectrum antibiotics, hydration and permanent capecitabine discontinuation	Full recovery after a few days of hydration and antibiotics
Mokrim, 2014 (case 2) [25]	67 y.o. woman with stage IV breast cancer	Palliative capecitabine	N.R.	/	Diarrhea, fever, nausea, fatigue	After 2nd cycle	CT: ileal walls thickening	Negative (tested afterwards)	Broad-spectrum antibiotics, hydration, bowel rest and permanent capecitabine discontinuation	Full recovery after a few days of hydration and antibiotics
Lee, 2015 (case 1) [26]	61 y.o. woman with stage IV colon cancer	Palliative capecitabine/irinotecan/bevacizumab	N.R.	/	Abdominal pain, diarrhea, vomit, G3 neutropenia, hypokalemia	After 4th cycle	CT: submucosal ileal edema, increased fat stranding	N.R.	Broad-spectrum antibiotics, hydration and permanent capecitabine discontinuation	Clinical recovery after 12 days of supportive therapy and dietary modifications
Lee, 2015 (case 2) [26]	59 y.o. woman with pT3N0 sigmoid colon cancer	Adjuvant capecitabine	2500 mg/m^2^ d1-14q21	/	Mucositis, hand-foot syndrome, diarrhea, abdominal pain, febrile neutropenia.	At 1st cycle, worsened after 3rd cycle	CT: submucosal ileal edema with fat stranding, pneumatosis intestinalis	N.R.	Broad-spectrum antibiotics, IV nutrition, electrolyte replacement and capecitabine discontinuation (not reported if restarted)	Clinical recovery after 29 days of supportive therapy and IV nutrition
Nicosia, 2017 (case 1) [27]	71 y.o. woman with cT3N1 lower rectal cancer	Neoadjuvant capecitabine + concurrent pelvic radiotherapy	825 mg/m^2^ twice daily	45 Gy/25 fx to pelvic nodal stations (bilateral common/internal iliac, presacral and obturator) 55 Gy/25 fx to rectum + mesorectum	Abdominal pain, diarrhea, vomit, hand-foot syndrome	After 16th fraction	CT: distal ileal edema with lumen reduction and small bowel distension	N.R.	Broad-spectrum antibiotics, hydration and permanent capecitabine discontinuation	Clinical recovery after 15 days of supportive therapy and antibiotics. Neoadjuvant treatment restarted with sole RT
Nicosia, 2017 (case 2) [27]	54 y.o. woman with cT3N0 lower rectal cancer	Neoadjuvant capecitabine + concurrent pelvic radiotherapy	825 mg/m^2^ twice daily	N.R.	Abdominal pain, dehydration, sub-occlusion	3 days after completion of RT-CHT	CT: ileal walls thickening with bowel loops distension and perivisceral effusion	N.R.	Broad-spectrum antibiotics, bowel rest with IV hydration and nutrition	Clinical recovery after 12 days of supportive therapy and antibiotics
Van Hellemond, 2018 [28]	69 y.o. woman with pT3N2 sigmoid colon cancer	Adjuvant capecitabine/oxaliplatin	N.R.	/	Nausea, appetite reduction, diarrhea and increased CRP	At CHT start	Colonoscopy w/biopsy: terminal ileitis with extensive inflammation. MR enterography; colic distension and thickening of the terminal ileal loop	Negative	Hydration and electrolyte replacement, antidiarrheal therapy, anti-inflammatory therapy and switch to FOLFOX	Clinical recovery with symptomatic therapy (duration of treatment not reported)
Dao, 2019 (case 1) [29]	72 y.o. woman with stage IIIC ascending colon cancer	Adjuvant capecitabine	N.R.	/	Diarrhea and G3 leuko-neutropenia	N.R.	CT: mild ileal loops dilation with vasa recta engorgement and mesenteric edema. Colonoscopy w/biopsy: granular erythematous mucosa and mucosal erosion	N.R.	Hydration and electrolyte replacement, broad-spectrum antibiotics, IV nutrition, anti-inflammatory therapy, antidiarrheal therapy and permanent capecitabine discontinuation	Persistence of symptoms for a total of four weeks after CHT discontinuation and supportive therapy initiation
Dao, 2019 (case 2) [29]	42 y.o. woman with recurrent breast cancer	Palliative capecitabine	N.R.	/	Abdominal pain, fever and bloody diarrhea with anemia and hypokalemia	N.R.	CT: ileal walls thickening and fluid filled bowel loops. Colonoscopy w/biopsy: terminal ileum with diffuse pseudomembranes, inflammatory exudates and spontaneous bleeding	N.R.	Hydration, broad-spectrum antibiotics, antidiarrheal therapy and permanent capecitabine discontinuation	Clinical resolution after four weeks of supportive therapy and antibiotics
Klimko, 2021 [30]	68 y.o. man with locally advanced colon cancer	Adjuvant capecitabine	N.R.	/	Diarrhea, nausea, vomit and malaise	10 days after CHT start	CT: ileal walls thickening. Colonscopy: large ileal ulcers and erythematous mucosa	(DPYD) *2A heterozygous mutation (tested afterward)	Hydration, antidiarrheal drugs and permanent capecitabine discontinuation	Clinical improvement three days after CHT discontinuation and symptomatic treatment
Zou, 2021 [31]	63 y.o. man with pT4N0 colon cancer	Adjuvant capecitabine/oxaliplatin	1000 mg/m^2^ twice daily	/	Bloody diarrhea with anemia and hypovolemic shock, fatigue	After 1st cycle	Colonoscopy: large amount of ileal bloody fluid. CT: submucosal ileal edema and fat stranding	Not performed	Emergency terminal ileal resection and permanent capecitabine discontinuation	Bloody diarrhea resolved after surgery
Gomez-Paz, 2022 [32]	69 y.o. man with colon cancer (stage N.R.)	Adjuvant capecitabine	N.R.	/	Watery diarrhea, pallor, abdominal pain and haematochezia. BTs: severe normocytic anemia, increased WBC count, hypokalemia, high lactate and increased INR	After 3rd cycle	Colonoscopy w/biopsy: erythematous mucosa and non-bleeding ulcerations from terminal ileum to ileo-colonic anastomosis	N.R.	Supportive care and capecitabine discontinuation (not reported if restarted)	Symptoms improvement with supportive care (duration of treatment not reported)
Sinha, 2022 [33]	42 y.o. woman with stage III (pT3N2) sigmoid colon cancer	Adjuvant capecitabine	N.R.	/	Abdominal pain and watery, bloody-tinged diarrhea	2 days after CHT start	CT: small bowel walls thickening most prominent in ileum with reactive edema. Colonoscopy w/biopsy: erythematous and friable mucosa with ulceration and exudate	Negative	IV antibiotics and permanent capecitabine discontinuation. Switch to different CHT agent (not specified) after discharge	N.R.
Shao, 2022 [34]	68 y.o. man with stage IIIB (pT3N1c) rectal cancer	Adjuvant capecitabine/oxaliplatin (switched to capecitabine monotherapy after 3 cycles due to recurrent G3 thrombocytopenia)	1500 mg twice daily d1-14q21	/	G3 diarrhea	During 2nd monotherapy cycle	CT/MRI: ileum and colon walls thickening and edema. Colonoscopy w/biopsy: hyperemia, patchy erosions and scattered ulcers	Variants of DPYD *5, DPYD *9A, TYMP and ABCB1	Symptomatic treatment and progressive capecitabine dose reduction. CHT discontinuation after 6 monotherapy cycles	Clinical resolution 2 weeks after capecitabine discontinuation
Our case	71 y.o. woman with cT3N2 rectal cancer	Neoadjuvant capecitabine + concurrent pelvic radiotherapy	825 mg/m^2^ twice daily	45 Gy/25 fx to pelvic nodal stations (bilateral internal iliac, presacral and obturator). 50 Gy/25 fx to rectum + mesorectum	Diarrhea, nausea, abdominal pain	After 17th fraction	CT: ileal walls thickening, mucosal hyperemia and vascular enhancement	Negative	Broad-spectrum antibiotics, bowel rest with IV nutrition and hydration, anti-inflammatory therapy, antidiarrheal therapy and permanent radiochemotherapy discontinuation. Capecitabine re-initiated at reduced dose in the post-operative setting	Clinical resolution after 20 days of supportive therapy and RT-CHT discontinuation

## Data Availability

Not applicable.

## Supplementary Materials

The following supporting information can be downloaded at: https://www.mdpi.com/article/10.3390/curroncol30100655/s1, Figure S1: PRISMA workflow of the literature search and article selection process.
