# Supplementary figures and images for: Capecitabine-Induced Ileitis during Neoadjuvant Pelvic Radio-Chemotherapy for Locally Advanced Rectal Cancer: A Case Report with Literature Review
Source: Curr Oncol. 2023 Oct 10;30(10):9063–77. doi: 10.3390/curroncol30100655 (PMC10605187; doi:10.3390/curroncol30100655)

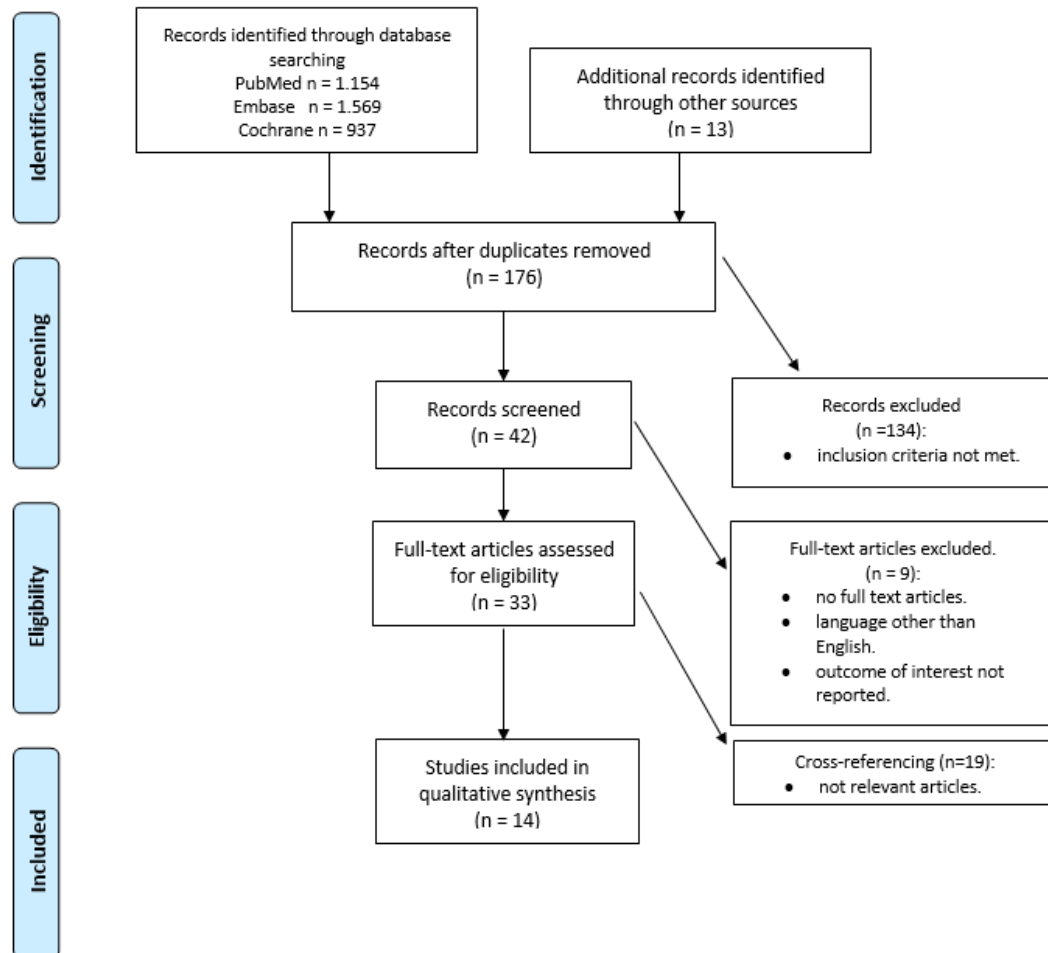

Figure S1: PRISMA workflow of the literature search and article selection process.

Supplement: Supplementary file 1 [file curroncol-30-00655-s001.zip › curroncol-2656283-supplementary.pdf]
